# Non‐Communicable Diseases (NCDs) in developing countries: a symposium report

**DOI:** 10.1186/s12992-014-0081-9

**Published:** 2014-12-11

**Authors:** Sheikh Mohammed Shariful Islam, Tina Dannemann Purnat, Nguyen Thi Anh Phuong, Upendo Mwingira, Karsten Schacht, Günter Fröschl

**Affiliations:** Center for International Health, Ludwig-Maximilians-Universität, Munich, Germany; Center for Control of Chronic Diseases (CCCD), icddr, b, Dhaka, Bangladesh; Hue University of Medicine and Pharmacy, Hue City, Vietnam; National Institute for Medical Research (NIMR), DRCP, Ministry of Health, Dar es Salaam, Tanzania

**Keywords:** Non-communicable diseases (NCDs), Developing countries, Symposium

## Abstract

In recent years, non-communicable diseases (NCDs) have globally shown increasing impact on health status in populations with disproportionately higher rates in developing countries. NCDs are the leading cause of mortality worldwide and a serious public health threat to developing countries. Recognizing the importance and urgency of the issue, a one-day symposium was organized on NCDs in Developing Countries by the CIH^LMU^ Center for International Health, Ludwig-Maximilians-Universität, Munich on 22nd March 2014. The objective of the symposium was to understand the current situation of different NCDs public health programs and the current trends in NCDs research and policy, promote exchange of ideas, encourage scientific debate and foster networking, partnerships and opportunities among experts from different clinical, research, and policy fields. The symposium was attended by more than seventy participants representing scientists, physicians, academics and students from several institutes in Germany and abroad. Seven key note presentations were made at the symposium by experts from Germany, UK, France, Bangladesh and Vietnam. This paper highlights the presentations and discussions during the symposium on different aspects of NCDs in developing countries. The symposium elucidated the dynamics of NCDs in developing countries and invited the participants to learn about evidence-based practices and policies for prevention and management of major NCDs and to debate the way forward.

## Background

In recent years, non-communicable diseases (NCDs), such as cardiovascular diseases (CVD), diabetes, chronic obstructive pulmonary diseases (COPD) and cancers have become an emerging pandemic globally with disproportionately higher rates in developing countries [1]. The World Health Organization (WHO) estimates that by 2020, NCDs will account for 80 percent of the global burden of disease, causing seven out of every 10 deaths in developing countries, about half of them premature deaths under the age of 70 [2-5]. According to WHO, it is estimated that the global NCD burden will increase by 17% in the next ten years, and in the African region by 27% [5]. Almost half of all deaths in Asia are now attributable to NCDs, accounting for 47% of global burden of disease [5]. Over 80% of cardiovascular and diabetes deaths, 90% of COPD deaths and two thirds of all cancer deaths occur in developing countries [6]. The transition from infectious diseases to NCDs in LMICs has been driven by a number of factors, often indicative of economic development: a move from traditional foods to processed foods high in fat, salt and sugar, a decrease in physical activity with sedentary lifestyles, and changed cultural norms such as increasing numbers of women using tobacco [7]. The impact of globalization and urbanization in low-and-middle-income countries (LMICs) has accelerated the growing burden of NCDs. However, governments in LMICs are not keeping pace with ever expanding needs for policies, legislation, services and infrastructure to prevent NCDs and poor people are the worst sufferers [8].

NCDs are a barrier to development [9]. In LMICs, poverty exposes people to behavioral risk factors for NCDs and in turn, resulting NCDs become an important driver for poverty [10]. The socioeconomic impacts of NCDs are also affecting progress towards the Millennium Development Goals (MDGs) [8] with serious implications for poverty reduction and economic development. Recognizing the importance and urgency, The United Nations High-level Meeting on the Prevention and Control of NCDs was organized in September 2011 [11]. The WHO advocates policy makers to develop efficient strategies to halt ‘tomorrow’s pandemic’ of the chronic NCDs [9]. In November 2012, WHO member states formally agreed on a comprehensive Global Monitoring Framework (GMF) for NCDs and the Global Action Plan for NCDs 2013–2020 (GAP) was formally agreed at the World Health Assembly in May 2013 [5,12,13]. A more concerted, strategic, and multi-sectorial policy approach is essential to help reverse the negative trends of NCDs in LMICs [14]. In this backdrop, the CIH^LMU^ Center for International Health at the Ludwig-Maximilians-Universität Munich (LMU) hosted a symposium on NCDs in developing countries as a part of a series that runs since 2010 contributing towards global health priorities by students of the PhD Program Medical Research – International Health as part of the programs regular curriculum. The objective of the symposium was to understand the current situation of various NCDs public health programs and the current trends in NCDs research and policy, promote exchange of ideas, encourage scientific debate and foster networking, partnerships and opportunities among experts from different clinical, research, and policy fields.

### Main text

The international symposium on “Non‐Communicable Diseases in Developing Countries” was held on March 22, 2014 at the Ludwig-Maximilians-Universität Munich, Germany. More than seventy participants representing scientists, physicians, academics and students from several institutes in Germany and abroad attended the symposium. The symposium covered seven technical presentations on different aspects of NCDs in the developing countries with enriching discussions by speakers from Bangladesh, France, Germany, Vietnam and the UK. During the symposium PhD students from LMU and other academic institutes conducted poster presentations on NCDs and other global health priority topics.

Dr. Shariful Islam from CIH^LMU^ and Senior Research Investigator, Center for Control of Chronic Diseases (CCCD), icddr,b opened the symposium on behalf of the organizing committee and stressed the recent global epidemic of NCDs affecting both the developed and developing nations. He stated that NCDs cause the largest mortality worldwide, accounting for 60% of global deaths. More than 80% of these deaths occur in LMICs, making NCDs a major cause of poverty and an urgent development issue. Dr. Islam stressed that NCDs strangle macro-economic development and keep the bottom billion locked up in chronic poverty. NCDs have a severe social and economic impact on individuals, communities and nations as a whole. The magnitude and rapid spread of NCDs means, we are all headed for a sick future unless we take action now. Thus, NCDs pose a double burden of disease in most LMICs where the health systems are least equipped to face the growing challenges. Prof. Dr. Thomas Löscher, Director, Department of Infectious Diseases and Tropical Medicine, Ludwig-Maximilians-Universität, Munich, Germany welcomed all participants and speakers to the symposium and highlighted the fact that new funding initiatives such as the Global Fund had led to great success with communicable diseases, drawing a parallel to new demands in the current situation of declining communicable diseases and increasing NCDs.

### Introduction to global epidemiology of NCDs and their control measures

Dr. Richard Smith, Former Editor, British Medical Journal, and President, United Health Group, UK.

### Summary presentation

The NCDs of global attention are CVD, diabetes, COPD and cancers. Tobacco use, poor diet, physical inactivity and alcohol are the four most common modifiable risk factors for NCDs. Mental health had only recently been included by the WHO as a NCD. The worsening burden of NCDs in the LMICs often comes accompanied by other factors straining health of the public. Results from Bangladesh data shows that during 1986–2006, deaths from NCDs increased from 8% to 68% in a rural area [15]. The decreasing trend of CVD in USA since 1980 is a testament to the fact that even increasing trend of NCDs as insurmountable as they are can be reversed. The three levels of causes for NCDs include underlying drivers, behavioral risk factors and metabolic, physiological risk factors. The challenge is to define an appropriate level for intervention. In developed countries most resources are at the last level and a rethink of strategy is necessary especially for LMICs. A holistic approach which addresses all of the three levels is required and there is a need for cooperation from different sectors including a complete government wide action on risk factors, sustained primary health care (PHC) with priority packages, surveillance and monitoring and learning from the integration of other programs such as HIV/AIDS. Measuring the impact of intervention for NCDs is difficult due to non-availability of reliable data for the targets. A Global Monitoring Framework for NCDs approved in May 2013 would be presented to the UN in September 2014 and the development of Sustainable Development Goals (SDGs) in 2015. Dr. Smith emphasized the fact that community based public health interventions, low and medium complexity interventions and screening all show reductions in Disability Adjusted Life-Years (DALYs), therefore representing candidates for best buys in reducing NCDs. Access to drugs is poor for some essential drugs in LMICs. The interwoven nature of NCDs and sustainable human development has implications for social, economic and environmental development. The rapidly increasing levels of CO_2_ emissions around the globe need to be addressed. The big effects on health in the future would come from malnutrition, extreme weather events (flooding and droughts), water shortages, mass migration and wars over resources. Policies that address climate change (less pollution, motorized transport, and meat production) are good for NCDs and vice versa. Sustainable agriculture and food production (more fruit and vegetables, less meat) mean more food, healthier food, less hunger and NCDs, and better income for rural farmers.

## Discussion

Mental health, occupational health and traumatology have been left out in many countries and on the agenda of WHO. People’s workplaces would be a good location to intervene for NCDs centered around preventing disease and improving healthy lifestyles. A consistent strategy for preventing mental illness is yet not in reach, and road traffic accidents need to be thought about seriously. It is necessary to identify policies that would deliver the greatest benefit across several NCDs. The general practitioners and schools need to be actively involved in the education under a whole society approach, creating environments where healthy choices are the easy choices.

### Economics of NCDs and Universal Health Coverage

Prof. Louis Niessen, Professor and Chair, Department of Health Economics, Liverpool School of Medicine and Tropical Medicine, UK.

### Summary presentation

Health economics have guided to set the health agenda in many countries by identifying the most cost-effective interventions for health. There is a decline in standardized death rates from heart disease around the world. The greatest absolute increase in population by 2030 would be in Africa (66%). This would mean that more numerous people are in need of care for NCDs. Health economics in public health helps in understanding how societies and individuals respond and how health is traded against other goods. Health economics has a role in promoting health and equity in health. Three aspects in health are important here: survival, quality of survival and people’s wants. Universal Health Coverage (UHC) is one of the contributions of health economics to health. UHC is about balancing efficiency and equity but there are always trade-offs to be made. UHC has become a global ambition and health systems require changes to achieve UHC and need to take structural barriers into account, and require political, financial and technical investments. Clinical and economic impacts are the most important factors for policy decisions and patient preferences. There is a global shift from emphasis on efficiency of interventions to equity of interventions. Elicitation methods are used across several countries to make decisions about resource allocations for health. Many countries choose efficiency over equity. Economic analyses strengthen the evidence base of priority setting at national or local level, and multi-criteria approaches are necessary and need further development.

## Discussion

Resources allocations for health should be based on individual country needs. Rational choices are even more critical in times of economic downturn as people’s willingness to pay for health is different. Health always ranks at the top of priority lists for households even in LMICs.

### Current research and programs for NCDs in low and middle income countries

Dr. Dewan Alam, Acting Director, Center for Control of Chronic Diseases (CCCD), icddr,b, Bangladesh.

### Summary presentation

Most NCD deaths are preventable and health systems are inadequate or unprepared or non-responsive to combat the threat of NCDs in most developing country settings. In Bangladesh, obesity levels are relatively low with most people physically active. However, harmful use of alcohol and smoking are very high in some LMICs. Importantly, most of these countries are witnessing an epidemiologic transition, yet are still facing widespread poverty. Data from Bangladesh shows that calorie intake levels were the 2nd lowest in the world. However, in South East Asia region abdominal obesity is very high even in people with low BMI. A third of the population in Bangladesh (218 million) would be over 60 years by 2050 which guarantees a high dependency ratio and high NCD burden. There has been a nine fold increase in deaths from NCDs in 20 years even though crude death rates have been stable throughout this period. Awareness about hypertension and blood pressure status among patients in Bangladesh is very low. Hypertension is higher in urban areas and even when diagnosed is difficult to control. Type 2 diabetes in Bangladesh reduces life by 6 years and will increase in all age groups. Abdominal obesity is a key effect modifier in Bangladesh. Two-thirds of COPD patients never knew they had irreversible lung condition and the prevalence is higher in rural areas compared to urban and is attributable to smoking and occupational exposures from cotton and jute industry. Solid fuel use and smoking coincide with the prevalence of COPD. About 50% of adult males in Bangladesh smoke tobacco. Women are exposed to indoor air pollution and also use smokeless tobacco. About 45,000 deaths are attributable to smoking in Bangladesh. Lifestyle modification interventions are more effective than metformin for reducing the incidence of diabetes. In Bangladesh, national surveillance and monitoring of NCDs have not been established yet.

### NCDs and the environment

Dr. Alexandra Schneider, Head of Research Group Environmental Risks, Helmholtz Zentrum Munich, Germany.

### Summary presentation

Air pollution and its impact on health is the area of research interest for Dr. Schneider. Smaller and finer particles are more harmful because they can find their way deeper into the lungs. Air pollution has effects on all systems of the body. There are three main pathways through which pollutants execute their harmful effects: 1) through pulmonary oxidative stress and inflammation, 2) autonomous nervous system imbalances and 3) particulate matter or constituents in the circulation. A causal relationship between PM2.5 (particulate matter of a size up to 2.5 μm) exposure and CVD morbidity and mortality exists. A study from Beijing in the years 2004–2005 showed an association between PM2.5 and CVD mortality and emergency room (ER) visits. Environmental interventions in China demonstrated it was possible to change environment effect, but these were short-lived. Not much research has been done in developing countries. One study estimates the burden of premature deaths and DALYs as 1.04 million and 31.4 million respectively. Increased temperature has serious effects on mortality and higher ER visits. Heat waves occur at very short time lag and have very pronounced effects on respiratory mortality. Cold effects last up to 4 weeks and a decrease in 1°C temperature increases mortality. In a Bangladeshi study all-cause mortality was highest in adult males, high socioeconomic status and in urban areas [16,17].

## Discussion

Optimal thermal ranges vary from one geographical location to another and populations have adapted to the climate. Also the effects of manufactured nano-particles on the lungs and circulation are to be considered. Health effects depend what material is loaded in the nano-particles. Current opinion is that ultra-fine particles are more dangerous than manufactured nano-particles and a direct effect on the autonomous nervous system and systemic inflammation can take place within 1–3 days. Health effects of moving between hot and cold rooms and cities should have similar effects on health. Air pollution might have an effect on cancers, however, beyond a certain threshold level of ambient air pollution there can be no further adverse effects on health.

### Epidemiology and prevention of cancer in the poorest countries

Dr. Silvia Franceschi, Special Advisor, Head of Infections and Cancer Epidemiology Group, International Agency for Research on Cancer (IARC), Lyon, France.

### Summary presentation

The age-standardized death rates from CVD are higher globally than in high-income countries (HICs). Malignant neoplasms accounted for 7.87 million deaths globally in 2011 and ranks number one in HICs. Cancer figures from the GLOBOCAN 2012 report provides estimates of the incidence, mortality and prevalence from major types of cancer globally [18]. More than half of all cancers (57%) and cancer deaths (65%) in 2012 occurred in LMICs, and these proportions will increase further by 2025. Incidence of cancer has been increasing in most regions of the world, but there are huge inequalities between rich and poor countries. Incidence rates remain highest in more developed regions, but mortality is much higher in less developed countries due to a lack of early detection and access to treatment facilities. From historical data, breast cancer rates increase as cervical cancer rates decline but data from Uganda showed an increase in both rates, which might be due to the prevalent HIV co-infection. Interventions in the essential package on global NCD targets vary from country to country depending on the dominant risk factors and vaccination can be an effective tool against a very heterogeneous group of diseases. Vaccination, screening and selected treatments like tamoxifen are simple to deliver even in LMIC settings. Vaccination can be one of the effective of the interventions. About 30% of infection-attributable cancer cases occur in people younger than 50 years. Increased wealth has been associated with a decreasing proportion of cancers attributable to infection. The important infections associated with cancer are Hepatitis B and C, Human Papiloma Virus (HPV) and Helicobacter pylori which accounts for 85-90% gastric cancers. Virus-like-particles-based vaccines have >90% efficacy in preventing HPV 16/18- related cancers which are responsible for between 68% and 82% of cervical cancers globally. HPV vaccines are currently too expensive for global recommendations considering $15/injection (3 doses required, studies showed that two doses might suffice). At $4.5/injection, it would be affordable and cost-effective to introduce the vaccine in GAVI countries (Global Alliance for Vaccines and Immunization). Data from two school-based projects in Bhutan and Rwanda shows >90% HPV vaccination coverage was attained. However, there is a need for careful articulation of the term ‘cancer-control’ and a reassessment of essential medicines and technologies in LMICs.

## Discussion

Efficacy of HPV vaccine has been demonstrated with a good proxy outcome for in situ carcinoma of the cervix. One dilemma is that it is not possible to prove efficacy against cervical cancer because of low incidence in HICs to demonstrate an effect. Also in LMICs, long follow-up (40 years) will be required to show the efficacies of vaccines. Smoking is also a major risk factor for cancers and more cancers can be prevented by evidence-based prevention programs. There is a need for policies on the early diagnosis of cancer in LMICs especially when the prerequisites for widespread screening programs have not been met.

### Diabetes research in developing countries

Dr. Andreas Lechner, Senior Physician, Diabetes Research Group, Medizinische Klinik und Poliklinik IV, Ludwig‐Maximilians‐Universität, Munich, Germany.

### Summary presentation

Type 2 diabetes mellitus (T2DM) is a complex disease with estimates of 382 million cases and similar number of undiagnosed cases. The costs diabetes in 2013 globally was 548 billion USD. About 80% of people with diabetes live in LMICs and a majority of them remains undiagnosed. The problem with T2DM is the long lag between disease cause and consequences such as blindness, kidney failure, heart attack and amputation. The reality of public programs is facing an enormous and growing number of affected people involving huge economic costs. T2DM results from an imbalance between insulin requirements and insulin secretion and shows strong genetic predisposition and also relationship with the environment. A study published in Nature Genetics on genome-wide association studies (GWAS) identified 62 variants but they are poor predictors of diabetes (predict only 5.7% of assumed genetic disposition). Lifestyle factors contributing to T2DM are obesity, low physical fitness, stress/insufficient sleep, rapid life transition, diet and smoking and also epigenetic factors such as birth weight and childhood nutrition. The probable pathogenic pathways of T2DM involve the adaptive tissue, muscle and brain. Although T2DM takes many years to develop, metabolic changes developed over many years prior to T2DM onset and could be monitored to delay progression. Gestational diabetes is an indicator of increased risk of T2DM. Crude estimates comparing Bangladeshi T2DM patients to Bavarian T2DM patients recruited at the same time in both countries showed that the Bangladeshi cohort had younger patients, with lower BMI but higher HbA1c levels than the German cohort [19]. This implies higher insulin resistance at the same age, more central obesity at young age, lower level of physical activity and higher intake of refined carbohydrates in Bangladeshi cohort. Interventions in patients with established T2DM do not help much in disease control and delaying onset of the disease should be priority. Lifestyle interventions can delay progression to T2DM by more than 5 years. There is a need for better understanding of the pathophysiology on a global scale, development of screening and prevention tools for LMICs and the testing of realistic tools to improve diagnosis in resource-poor settings.

## Discussion

Weight reduction is the key to prevention and management of diabetes regardless of dietary constituents. However, the thresholds for weight loss in various populations are not defined. The disparity in outcome between T2DM patients in Germany and Bangladesh could be due to early diagnosis in Germany and medication adherence. Metformin has been shown to work for pre-diabetes and delay of the onset of T2DM. However, its use for pre-diabetes is not approved in Germany.

### Health systems and NCDs in developing countries: experience from Vietnam

Prof. Tran Huu Dang, Former Vice-Rector, Internal Medicine Department, Hue University of Medicine and Pharmacy, and Vice President of Vietnamese Association of Diabetes and Endocrinology, Vietnam.

### Summary presentation

Increased life expectancy in Vietnam has led to increased exposure to NCD risk factors. Mortality due to NCDs is four times higher than that inferred by infectious diseases and morbidity is more than 62%. CVD is the leading cause of death in Vietnam. Stroke and depression contribute most to DALYs in males and females respectively in Vietnam. About one-fourth of adults are hypertensive and more than half (5.7 million) remain undiagnosed. Each year there are 200,000 new stroke patients and 11,000 deaths; 250,000 people with cancer with 75,000 deaths. More than 60% of the population are smokers. No age restrictions exist on the sale of tobacco and tax on cigarettes is very low. Vietnam has the highest COPD prevalence in Asia (6.7%). The prevalence of diabetes is 5.7%, 60% remain undiagnosed and only 20% are treated and controlled. Vietnam has a 3 tier health system but utilization of the PHC is very low due to underfunding. A large number of people bypass the PHC and go to secondary level provincial hospitals directly leading to increased costs. There is a new initiative at the Department of Family Medicine, Hue University to train family medicine and paramedical personnel posted to PHC level facilities and communities in order to meet the increasing demand. The next step would be a scaling up of the training program to all provinces of Vietnam and a national proposal to reduce overloading at hospitals is on-going (2012–2020).

## Discussion

Doctors completing 6 years of medical training do currently an additional 2 year curriculum in family medicine. About 100 doctors have graduated as family medicine specialists in Vietnam. A shift in healthcare work force towards paramedical personnel would increase the availability of human resources in health care considerably.

## Conclusions

The symposium provided the audience with a current update about NCD policies, research and programs globally, with debates about the priority action plans and suggestions for a way forward. Identifying cost-effective strategies and innovative measures like Universal Health Coverage should be considered by policy makers. Cessation of smoking, awareness building, screening and lifestyle interventions can be effective tools for prevention of cancers and diabetes in LMIC settings. Climate change and environment can have an impact on NCDs. Health systems in most developing countries are unprepared to deal with the burden of NCDs and developing a human resource for NCDs should be considered to provide prevention and management of NCDs at the primary health care settings.

From the above discussions we can conclude that NCDs are the largest health burden in LMICs and an all-out effort by governments and different stakeholders is needed to control this pandemic. As a particular aspect the event strengthened the capacity of participating young professionals to better understand NCDs and join the fight to combat the global NCD crisis. Symposiums on NCDs can work as a platform for networking between different organizations, individuals from different parts of the world and diverse specialties. We believe that the event was instrumental in sensitizing people on different aspects of NCDs in developing countries and in drawing the attention of current and future public health professionals.
